# Influence of endothelial function and arterial stiffness on the behavior of cervicocephalic arterial dissections: An observational study

**DOI:** 10.3389/fneur.2022.968488

**Published:** 2022-08-29

**Authors:** Seong-Joon Lee, Jin Soo Lee, Min Kim, So Young Park, Ji Hyun Park, Bumhee Park, Woo Sang Jung, Jin Wook Choi, Ji Man Hong

**Affiliations:** ^1^Department of Neurology, Ajou University School of Medicine, Suwon-si, South Korea; ^2^Office of Biostatistics, Medical Research Collaborating Center, Ajou Research Institute for Innovative Medicine, Ajou University Medical Center, Suwon-si, South Korea; ^3^Department of Biomedical Informatics, Ajou University School of Medicine, Suwon-si, South Korea; ^4^Department of Radiology, Ajou University School of Medicine, Suwon-si, South Korea

**Keywords:** cervicocephalic arterial dissection, endothelial function, arterial stiffness, flowmediated dilatation, pulse wave velocity

## Abstract

**Background:**

The mechanical and physiological properties of the arterial wall might affect the behavior of spontaneous cervicocephalic arterial dissections (CCAD). We aimed to determine the effects of endothelial function and arterial stiffness on the clinical characteristics and outcomes of CCAD using brachial flow-mediated dilation (FMD) and brachial-ankle pulse wave velocity (PWV).

**Methods:**

From a single-center database, we identified patients admitted from April 2011 to December 2021 with a diagnosis of CCAD who underwent both FMD and PWV. FMD was classified as normal and decreased according to institutional thresholds. PWV was categorized into tertiles. Comparative and multivariable analyses were performed to determine the effects of FMD and PWV values on major clinical outcomes.

**Results:**

A total of 146 patients (age: 47 ± 11 years; men: 77.4%) were included. The main presentation was ischemic stroke in 76.7% of the patients, while 23.3% presented with headache or other symptoms. Healing of the dissection was observed in 55.8%. In multivariable analysis, Normal FMD levels (vs. decreased; adjusted OR: 4.52, 95% CI [1.95 −10.52]) were associated with spontaneous healing of the dissection. Highest PWV tertile (vs. lowest; adjusted OR: 17.05, 95% CI [3.07–94.82]) was associated with ischemic presentation. There was a higher ischemic stroke recurrence in the 3rd PWV tertile, and more frequent aneurysmal enlargement in the lowest PWV tertile, but their frequency was low, precluding multivariable analysis.

**Conclusion:**

In spontaneous CCAD, preserved endothelial function was associated with spontaneous arterial healing. Arterial stiffness is associated with ischemic presentation.

## Background

The clinical behavior of cervicocephalic arterial dissection (CCAD) is dynamic. It can cause both ischemic and hemorrhagic stroke and is a leading cause of stroke in patients below 45 years of age, accounting for almost one-fourth of all cases of strokes in this population (1). They may also present with headache, neck pain, or signs of compression of cranial nerves. Unlike atherosclerotic diseases, which are static lesions, a dissecting arterial lesion tends to undergo morphological changes, and a decrease in the size of the dissecting aneurysm and resolution of luminal stenosis is observed in ~60% of patients with intracranial dissection of the vertebrobasilar artery (2). However, spontaneous healing is not universal. Some patients develop aneurysmal enlargement (3), which might be fatal if the aneurysm ruptures, resulting in subarachnoid hemorrhage. For ischemic stroke, there is currently no evidence that incomplete vascular healing increases future ischemic events. However, it is rational to infer that incomplete vascular healing might result in a longer duration of medical therapy, increased medical costs, and recurrent stroke.

The arterial wall exhibits physiological and mechanical properties. Its physiological properties are mainly defined by the endothelium, which is arguably the largest and most diffuse endocrine organ of the body (4). The endothelium also plays an important role in the healing of an arterial injury. Following the loss of endothelial cells due to vessel injury, endothelial regrowth occurs, and the quality and extent of its regrowth have important interactions with arterial thrombogenicity, neointimal hyperplasia, and vessel remodeling following arterial injury (5). It is plausible that differences in the endothelial function might alter the arterial response to spontaneous dissections. Endothelial function mediated by a nitric oxide-dependent mechanism can be easily evaluated by flow-mediated dilation (FMD), which is a non-invasive method for evaluating endothelial function through an increase in shear stress by reactive hyperemia (6). Endothelial dysfunction, as measured by FMD, is generally considered the earliest stage of atherosclerosis, and is a predictor of future cerebrovascular disease (7, 8). Alteration in endothelial function precedes morphological atherosclerotic changes and can lead to lesion development and clinical complications (9).

Among the mechanical properties of the arterial wall, arterial stiffness is an important parameter as a biomarker of arteriosclerosis and is also associated with end-organ injury (10). Arterial stiffness might also affect the arterial behavior following spontaneous dissections. Arterial stiffness can be evaluated by pulse wave velocity (PWV), which evaluates the artery's ability to expand and contract with cardiac pulsation and relaxation (11). There is little correlation between PWV and classical risk factors for atherosclerosis other than age and blood pressure, while it is associated with aortic calcification that occurs within advanced atherosclerotic plaque (7).

In this study, we hypothesized that endothelial function and arterial wall stiffness will be associated with behavior of arterial wall in response to vessel injury. In a single center CCAD population, we evaluated the association between FMD, PWV, and major clinicoradiologic outcomes.

## Methods

### Study population and management

**Figure 1:**
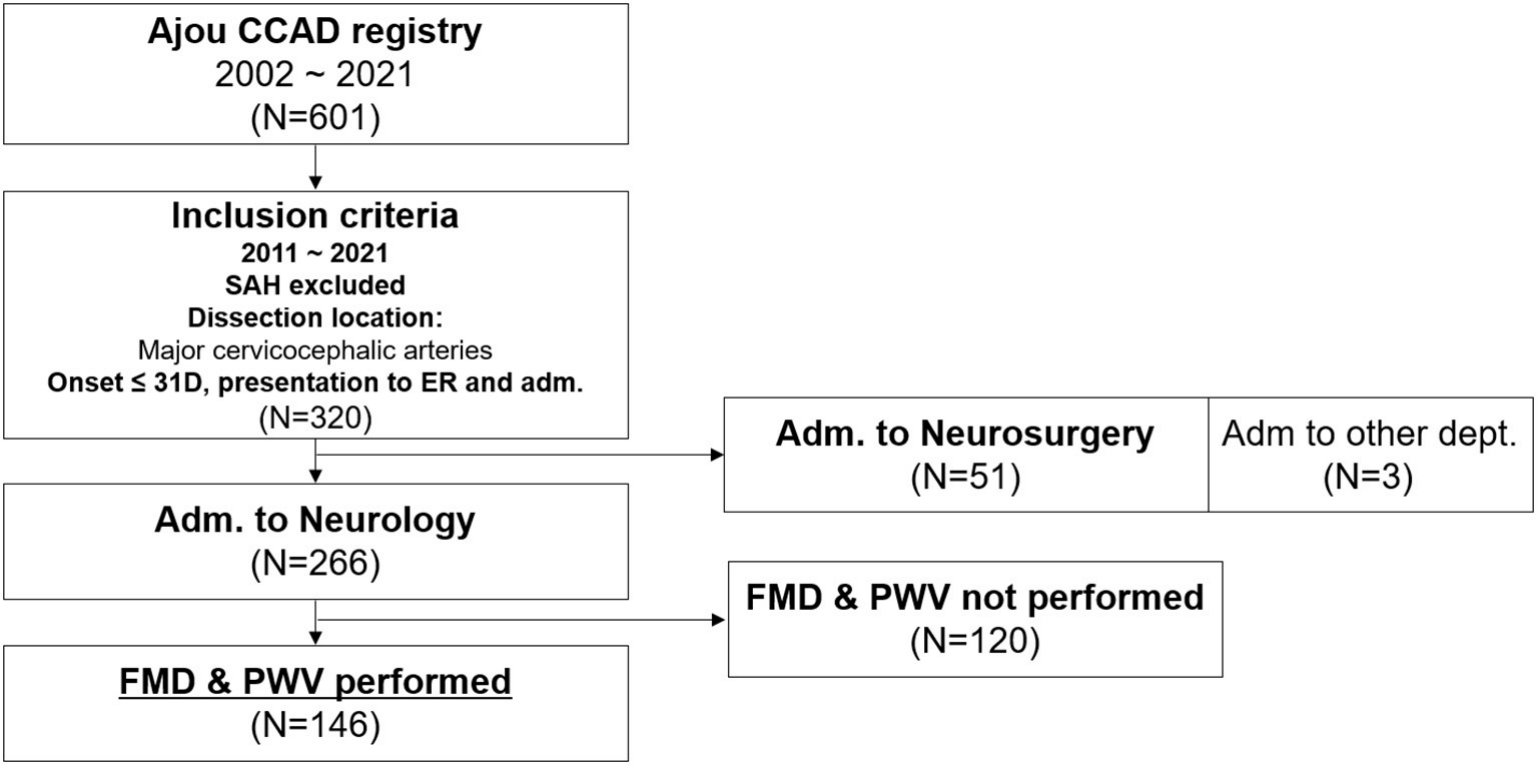
Flowchart of patient inclusion for the current study. CCAD, cervicocephalic artery dissection; SAH, subarachnoid hemorrhage; ER, emergency room; FMD, flow-mediated dilation; PWV, pulse wave velocity.

Data were collected by a retrospective medical record search. The hospital's electronic medical records were first searched for patients diagnosed with “dissection of intracranial, extracranial, or cerebral arteries, ruptured or unruptured.” Next, the neurology admission patient database was reviewed for the diagnosis of CCAD. Third, patients admitted with a diagnosis of subarachnoid hemorrhage were screened based on the term ‘dissection' in the imaging reports of the brain. The brain images of the patients were then reviewed by a researcher for the confirmatory diagnosis of CCAD (12). In this registry (n = 601), patients who met the below criteria were included for analysis; 1) patients who presented between April 2011 and December 2021, with the primary dissection nidus located at the major cervicocephalic arteries (extracranial and intracranial internal carotid artery and vertebral artery, up to the middle cerebral artery M2 and anterior cerebral artery A2 and the basilar artery to posterior cerebral artery), 2) exclusion of patients who presented with subarachnoid hemorrhage, 3) the patient presented to the emergency department with duration between the onset of symptoms to presentation within 31 days, 4) the patient was admitted to the neurology department for acute treatments, and 5) both FMD and PWV performed as part of routine work-up for recurrent cerebrovascular disease risk stratification, were selected for the analysis (Figure 1). Ethics approval was obtained from Ajou University Hospital IRB and have therefore been performed in accordance with the ethical standards laid down in the 1964 Declaration of Helsinki and its later amendments. The board waived the need for patient consent.

### Variables and image analysis

**Figure 2:**
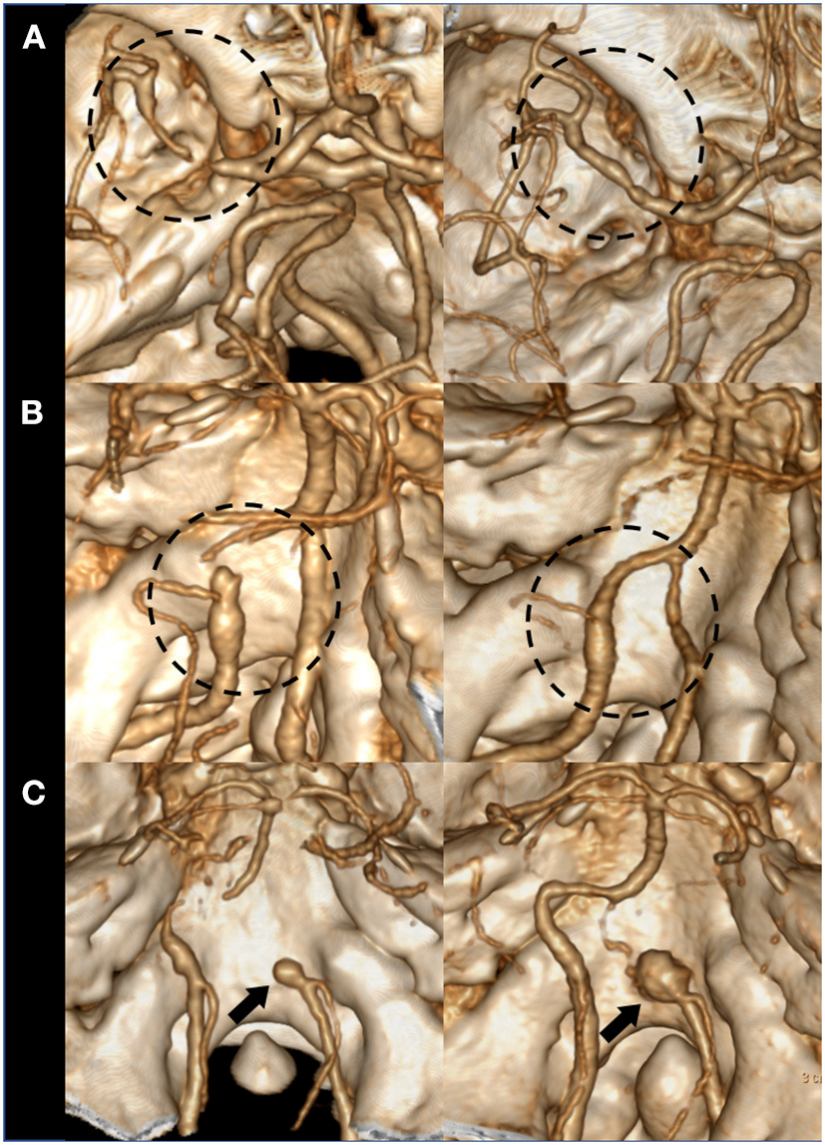
Examples of spontaneous arterial healing and aneurysmal changes. **(A)** An originally stenotic dissection segment shows arterial healing with improved stenosis degree. **(B)** A dissection segment with dilatation pattern (dilatation & stenosis) shows arterial healing with reduced aneurysm size and improved stenosis. **(C)** An initially dilated dissection segment shows aneurysmal enlargement on serial imaging.

The clinical presentation was classified as ischemic stroke (confirmation by diffusion weighted imaging), hemorrhagic stroke, or headache and other symptoms. Baseline demographics were collected. The primary arterial luminal morphology was described as stenosis pattern, dilatation pattern (including stenosis and dilatation), and occlusion pattern. The stenosis and dilatation pattern was classified as dilatation pattern according to a previous report that showed similarly higher rupture risk compared to steno-occlusive patterns in these two patterns (2). The location of the dissection was categorized into intracranial and extracranial (including extended extracranial to intracranial dissections, tandem extracranial dissection with intracranial occlusions). The primary treatment modality of the patient was classified as acute neurointervention, delayed neurointervention, bypass surgery, or medical management. In patients with ischemic presentation, the initial clinical severity was graded using the National Institute of Health Stroke Scale (NIHSS), which was measured 3 times daily during acute stroke unit care, and then daily until discharge. Early neurological deterioration (END) was classified as an increase of 2 or more points in the NIHSS score within 7 days post-admission (13). Recurrent ischemic stroke or recurrent subarachnoid hemorrhage events were identified. Functional outcomes were graded using the modified Rankin Scale (mRS) score at 3 months. Serial noninvasive angiographic images of the same modality were analyzed to evaluate arterial healing in patients not treated with early neurointervention. Computed tomography angiographic images were used for most patients because it is the primary imaging modality for patients presenting with cerebrovascular disease to our emergency department. Spontaneous arterial healing was classified as any improvement in the luminal diameter for stenotic or occlusive lesions and any decrease in the aneurysm size for dilatation patterns (2). Aneurysmal changes or increase in aneurysm sizes were specifically evaluated (Figure 2).

### Measurement of endothelial function and arterial stiffness

In all ischemic stroke and cerebrovascular disease patients admitted to the department of neurology in our hospital, brachial FMD and ankle-brachial artery PWV is usually evaluated as a second line routine work-up for recurrent cerebrovascular disease risk stratification and prevention, along with first line work up such as transthoracic echocardiography and 24 h holter monitoring, which is strongly recommended as stroke etiologic evaluation by current guidelines (14). It is usually performed after the 3 or 4 days of acute stroke management period, during admission.

Endothelial function was measured by FMD in the brachial artery using the post-ischemic forearm hyperemia method. Cross-sectional imaging with M-mode using a fixation device was used to manually measure the arterial diameter (15). FMD values were dichotomized (16), and FMD values ≤ 5.8% were classified as decreased endothelial function, according to institutional thresholds (lower than 1 standard deviation of FMD values of normal volunteers). Arterial stiffness was measured using brachial-ankle PWV (VP-1000, Omron, Kyoto, Japan). The mean value of right and left PWV was used, and the values were categorized according to tertiles (17).

### Statistical analysis

To evaluate the significance of FMD values on the behavior of CCAD, the study population was classified into decreased FMD and normal FMD groups. A comparative analysis was performed between the two groups. To evaluate the significance of PWV findings on the behavior of CCAD, the population was categorized according to the PWV tertile. Comparative analyses were performed for all three groups. Continuous variables were compared using the student's *t*-test and Mann–Whitney *U*-test, and categorical variables were analyzed using the chi-square test or Fisher's exact test. Based on the univariate analysis, the association between FMD and spontaneous arterial healing was evaluated using multiple logistic regression, including clinically significant co-variables and variables identified in univariate analysis. It was performed with FMD values in both dichotomized form and as a continuous variable. The association between PWV tertile and ischemic presentation was also evaluated using multiple logistic regression, including clinically significant co-variables. This analysis was performed with PWV values in both trichotomized form and as a continuous variable (PWV divided by 100 [PWV/100]). All statistical analyses were performed by use of R software, version 4.0.5. A *P*-value less than 0.05 was considered to be statistically significant.

## Results

Among 601 CCAD patients, a total of 320 patients presented to the emergency department with a CCAD involving the major cervicocephalic arteries during the study period and was admitted for acute management, excluding those who presented with subarachnoid hemorrhage. Among them, 51 were admitted to the neurosurgery department for concerns of dissecting aneurysmal rupture. Among 266 patients admitted to the neurology department, 146 patients performed both PWV and FMD, and were included in the analysis (Figure 1). Comparison of clinical characteristics and outcomes between patients admitted to the neurology department with FMD and PWV performed, those admitted to the neurology department but without FMD and PWV data, and patients that were admitted to the neurosurgery department is shown as Supplementary Table 1. A total of 146 patients (age: 47 ± 11 years; men: 77.4%) were included for the main analysis. The main presentation was ischemic stroke in 76.7% of the patients, while 23.3% presented with headache or other symptoms. Healing of the dissection was observed in 55.8%.

### FMD and its association between clinicoradiologic variables

When the patients were categorized into the decreased FMD group (*n* = 53; 36.3%) and normal FMD group (*n* = 93; 63.7%%) (Table 1), the decreased FMD group were older (51 ± 11 vs. 45 ± 10, *p* = 0.002) and were more frequently male (46/53 [86.8%] vs. 67/93 [72.0%], *p* = 0.040). There were no differences in symptom onset to presentation or symptoms onset to FMD evaluation. There were no differences in the presentation patterns, dissection luminal morphology, or anterior vs. posterior circulation predominance. Intracranial dissections were predominant in the decreased FMD group (43/53 [81.1%] vs. 52/93 [55.9%], *p* = 0.002), and a higher rate of comorbid hypertension was seen (28/53 [52.8%] vs. 30/93 [32.3%], *p* = 0.015). A lower proportion of patients in the decreased FMD group received anticoagulants (22/53 [41.5%] vs. 65/93 [69.9%], *p* = 0.003). The results of clinical follow-up did not show any difference between the two groups, with similar 3-month mRS, similar rates of new ischemic stroke, and no recurrent subarachnoid hemorrhage in each group. However, in the arterial healing analysis, spontaneous arterial healing was more common in the normal FMD group than in the decreased FMD group (57/81 [70.4%] vs. 15/48 [31.3%], *p* < 0.001). A multivariable analysis was performed to identify the association between FMD values and spontaneous arterial healing including clinically significant variables, and variables identified through the univariate analysis (Supplementary Table 2). In the multivariable analysis, a normal FMD (as compared to decreased FMD, dichotomized form) was associated with spontaneous arterial healing (odds ratio: 4.52, 95% confidence interval [1.95–10.52*], p* < 0.001) along with comorbid diabetes mellitus (OR: 0.07, 95% CI [0.01–0.70], *p* = 0.023), age, luminal morphology, and extracranial (vs. intracranial) location as co-variables (Table 2, Model 1). FMD values as a continuous variable was also associated with spontaneous arterial healing (OR: 1.37, 95% CI [1.06–1.76], *p* = 0.014) along with the same co-variables (Table 2, Model 2). Aneurysmal change was not associated with FMD.

**Table 1:** Baseline profiles, treatment, clinical, and imaging characteristics according to dichotomized state of flow mediated dilatation.

	**Decreased FMD** **(*N* = 53)**	**Normal FMD** **(*N* = 93)**	***P*-value**
Age	51 ± 11	45 ± 10	0.002
Sex, male	46 (86.8%)	67 (72.0%)	0.040
Onset to presentation, d	1 [0–3.5]	1 [0–5.5]	0.489
Presentation pattern			0.340
Ischemic stroke	43 (81.1%)	69 (74.2%)	
Headache, others	10 (18.9%)	24 (25.8%)	
Onset to FMD, d	9 [6–13]	10 [6–14]	0.860
Morphology			0.368
Stenosis	17 (32.1%)	20 (21.5%)	
Dilatation	16 (30.2%)	32 (34.4%)	
Occlusion	20 (37.7%)	41 (44.1%)	
Circulation			0.992
Carotid	16 (30.2%)	28 (30.1%)	
Vertebro-basilar	37 (69.8%)	65 (69.9%)	
Intra vs. extracranial			0.002
Intracranial	43 (81.1%)	52 (55.9%)	
Extracranial	10 (18.9%)	41 (44.1%)	
HTN	28 (52.8%)	30 (32.3%)	0.015
DM	5 (9.4%)	7 (7.5%)	0.687
Smoking	25 (47.2%)	35 (37.6%)	0.260
Dyslipidemia	13 (24.5%)	20 (21.5%)	0.675
Treatments			0.398
Medical	47 (88.7%)	81 (87.1%)	
Acute intervention	5 (9.4%)	10 (10.8%)	
Delayed intervention	1 (1.9%)	0 (0.0%)	
Bypass operation	0 (0.0%)	2 (2.2%)	
Medications			0.003
None	5 (9.4%)	3 (3.2%)	
Antiplatelet	26 (49.1%)	25 (26.9%)	
Anticoagulation	22 (41.5%)	65 (69.9%)	
**Clinical follow-up**	(*N* = 53)	(*N* = 90)	
3-month mRS	1.0 [1.0–2.0]	1.0 [0.0–1.0]	0.068
New ischemic stroke	5 (9.4%)	3 (3.3%)	0.125
New SAH	0 (0.0%)	0 (0.0%)	
**Image follow-up**	(*N* = 48)	(*N* = 81)	
Spont. arterial healing	15 (31.3%)	57 (70.4%)	<0.001
Aneurysm enlargement	3 (6.3%)	1 (1.2%)	0.112
**Ischemic course**	(*N* = 43)	(*N* = 69)	
NIHSS	3.0 [1.0–5.0]	2.0 [1.0–5.0]	0.875
END	7 (16.3%)	6 (8.7%)	0.223
3-month mRS	1.0 [1.0–2.0]	1.0 [0.0–2.0]	0.308

FMD, flow mediated dilatation; HTN, hypertension; DM, diabetes mellitus; mRS, modified Rankin Scale; SAH, subarachnoid hemorrhage; NIHSS, National Institute of Health Stroke Scale; END, early neurological deterioration.

**Table 2:** Multivariable analysis to determine the association between flow mediated dilatation values and spontaneous arterial healing.

**Variable**	**Odds ratio**	***P*-value**
**Model 1**		
Normal FMD (vs. decreased)	4.52 [1.95–10.52]	<0.001
Age	0.98 [0.94–1.02]	0.347
DM	0.07 [0.01–0.70]	0.023
Morphology		
Stenosis	Reference	
Dilatation	1.71 [0.60–4.93]	0.318
Occlusion	1.42 [0.49–4.10]	0.516
Extracranial (vs. intracranial)	1.51 [0.57–4.00]	0.411
**Model 2**		
FMD as a continuous variable	1.37 [1.06–1.756]	0.014
Age	0.98 [0.94–1.02]	0.278
DM	0.06 [0.01–0.64]	0.02
Morphology		
Stenosis	Reference	
Dilatation	1.94 [0.69–5.47]	0.21
Occlusion	1.58 [0.55–4.48]	0.394
Extracranial (vs. intracranial)	1.45 [0.55–3.81]	0.457

FMD, flow mediated dilatation; DM, diabetes mellitus.

### PWV and its association between clinicoradiologic variables

When the patients were categorized according to the PWV tertiles (Table 3), higher PWV tertial groups were older (40 ± 10 vs. 47 ± 8 vs. 54 ± 10. *p* < 0.001 [*Post-hoc* Bonferroni test, *p* < 0.005 for all inter-group comparisons]). The proportion of patients with ischemic presentation increased as PWV tertiles increased (32/49 [65.3%] vs. 35/50 [70.0%] vs. 45/47 [95.7%]), *p* = 0.001). While symptom onset to presentation time was shorter in 3^rd^ tertile group compared to 1^st^ tertile (days, 2 [0–6.5] vs. 1.5 [0–6] vs. 1 [0–2], *p* = 0.007), likely due to a higher ischemic stroke presentation, there were no differences in symptom onset to PWV evaluation. With increasing PWV tertiles, dilatation morphology decreased (23/49 [46.9%] vs. 13/50 [26.0%] vs. 12/47 [25.5%]) and occlusion morphology increased (12/49 [24.5%] vs. 26/50 [52.0%] vs. 23/47 [48.9%]) (*p* = 0.037). There was also a higher prevalence of comorbid hypertension (10/49 [20.4%] vs. 19/50 [38.0%] vs. 29/47 [61.7%], *p* < 0.001) with the increase in tertiles. In the clinical follow-up, there was a predominance of ischemic stroke recurrence in the 3rd tertile (1/48 [2.1%] vs. 0/50 [0.0%] vs. 7/47 [14.9%], *p* = 0.003). There were no differences in spontaneous arterial healing, while aneurysmal enlargement more frequently occurred in the lowest PWV tertile (4/44 (9.1%) vs. 0/44 (0.0%) vs. 0/41 (0.0%). *P* = 0.019). A multivariable analysis was performed to identify the association between PWV values and ischemic stroke presentation including clinically significant variables, and variables identified through the univariate analysis (Supplementary Table 3). In multivariable analysis, the third tertile of PWV was associated with ischemic presentation (first PWV tertile as a reference, OR: 17.05, 95% CI [3.07–94.82], *p* = 0.001), along with extracranial dissections (intracranial as a reference OR: 4.45, 95% CI [1.25–15.89], *p* = 0.021), age, sex, and dissection morphology as co-variables (Table 4). PWV as a continuous variable (PWV/100, PWV values divided by 100) was also associated with ischemic presentation (OR: 1.44, 95% CI [1.14–1.82], *p* = 0.002), along with the same co-variables (Table 4).

**Table 3:** Baseline profiles, treatment, clinical, and imaging characteristics according to the tertiles of pulse wave velocity values.

	**1st tertile (*N* = 49)**	**2nd tertile (*N* = 50)**	**3rd tertile (*N* = 47)**	***P*-value**
Age	40 ± 10	47 ± 8	54 ± 10	<0.001*
Sex, male	36 (73.5%)	37 (74.0%)	40 (85.1%)	0.307
Onset to presentation, d	2 [0–6.5]	1.5 [0–6]	1 [0–2]	0.007^†^
Presentation pattern				0.001
Ischemic stroke	32 (65.3%)	35 (70.0%)	45 (95.7%)	
Headache, others	17 (34.7%)	15 (30.0%)	2 (4.3%)	
Onset to PWV	11 [6–18]	10 [6–16]	8 [5–12]	0.198
Morphology				0.037
Stenosis	14 (28.6%)	11 (22.0%)	12 (25.5%)	
Dilatation	23 (46.9%)	13 (26.0%)	12 (25.5%)	
Occlusion	12 (24.5%)	26 (52.0%)	23 (48.9%)	
Circulation				0.879
Carotid	16 (32.7%)	14 (28.0%)	14 (29.8%)	
Vertebro-basilar	33 (67.3%)	36 (72.0%)	33 (70.2%)	
Intra vs. extracranial				0.809
Intracranial	32 (65.3%)	34 (68.0%)	29 (61.7%)	
Extracranial	17 (34.7%)	16 (32.0%)	18 (38.3%)	
HTN	10 (20.4%)	19 (38.0%)	29 (61.7%)	<0.001
DM	3 (6.1%)	3 (6.0%)	6 (12.8%)	0.387
Smoking	18 (36.7%)	22 (44.0%)	20 (42.6%)	0.741
Dyslipidemia	7 (14.3%)	13 (26.0%)	13 (27.7%)	0.228
Treatments				0.762
Medical	42 (85.7%)	44 (88.0%)	42 (89.4%)	
Acute intervention	6 (12.2%)	5 (10.0%)	4 (8.5%)	
Delayed intervention	1 (2.0%)	0 (0.0%)	0 (0.0%)	
Bypass operation	0 (0.0%)	1 (2.0%)	1 (2.1%)	
Medications				0.323
None	3 (6.1%)	4 (8.0%)	1 (2.1%)	
Antiplatelet	17 (34.7%)	13 (26.0%)	21 (44.7%)	
Anticoagulation	29 (59.2%)	33 (66.0%)	25 (53.2%)	
**Clinical follow-up**	(*N* = 48)	(*N* = 48)	(*N* = 47)	
3-month mRS	1.0 [0.0–1.0]	1.0 [0.25–2.0]	1.0 [0.0–2.0]	0.055
New ischemic stroke	1 (2.1%)	0 (0.0%)	7 (14.9%)	0.003
New SAH	0 (0.0%)	0 (0.0%)	0 (0.0%)	
**Image follow-up**	(*N* = 44)	(*N* = 44)	(*N* = 41)	
Spont. arterial healing	25 (56.8%)	28 (63.6%)	19 (46.3%)	0.272
Aneurysm enlargement	4 (9.1%)	0 (0.0%)	0 (0.0%)	0.019
**Ischemic course**	(*N* = 32)	(*N* = 35)	(*N* = 45)	
NIHSS	2.0 [1.0–4.0]	3.0 [1.0–5.0]	3.0 [1.0–5.0]	0.889
END	3 (9.4%)	4 (11.4%)	6 (13.3%)	0.866
3-month mRS	1.0 [0.0–1.0]	1.0 [1.0–2.0]	1.0 [0.0–2.0]	0.084

HTN, hypertension; DM, diabetes mellitus; mRS, modified Rankin Scale; SAH, subarachnoid hemorrhage; NIHSS, National Institute of Health Stroke Scale; END, early neurological deterioration.

*Post hoc Bonferroni test, p < 0.005 for all inter-group comparisons.

^†^Post hoc Bonferroni test, 1st tertile vs. 3rd tertile, p = 0.005.

**Table 4:** Multivariable analysis to evaluate the association between pulse wave velocity values and ischemic stroke presentation.

**Variable**	**Odds ratio**	***P*-value**
**Model 1**		
PWV tertile		
1^st^ tertile	Reference	
2^nd^ tertile	1.37 [0.46–4.04]	0.568
3^rd^ tertile	17.05 [3.07–94.82]	0.001
Age	0.97 [0.92–1.02]	0.202
Sex, male	2.28 [0.85–6.07]	0.102
Morphology		
Stenosis	Reference	
Dilatation	0.41 [0.14–1.22]	0.108
Occlusion	1.56 [0.43–5.68]	0.501
Extracranial (vs. intracranial)	4.45 [1.25–15.89]	0.021
**Model 2**		
PWV/100, continuous variable	1.44 [1.14–1.82]	0.002
Age	0.96 [0.91–1.01]	0.106
Sex, male	2.17 [0.82–5.75]	0.119
Morphology		
Stenosis	Reference	
Dilatation	0.41 [0.14–1.21]	0.106
Occlusion	1.42 [0.40–4.97]	0.586
Extracranial (vs. intracranial)	4.79 [1.34–17.1]	0.016

PWV, pulse wave velocity.

## Discussion

The results of our study show that preserved endothelial function is associated with spontaneous arterial healing in patients with CCAD, while arterial stiffness is associated with ischemic presentation. While ischemic stroke recurrence was more frequent with increases in arterial stiffness, and aneurysmal enlargement more frequent in the lowest tertile of arterial stiffness, the frequency of both recurrent ischemic stroke and aneurysmal enlargement was low, precluding further analysis.

To our knowledge, this is the first study to show that endothelial function is associated with arterial healing. Previously reported factors that influence arterial healing have largely focused on high resolution MRI morphology of the dissection segment (2, 18), which may represent temporal profile of the arterial dissections, or incomplete repair of vessel walls (3). Location and geometry (19) have been also described, such as C2 locations (18), lower posterior inferior cerebellar artery involvement and vertebrobasilar artery union angle (19), that might be associated with recurrent mechanical stress or hemodynamic properties. However, inverse relations with vascular risk factors such as smoking, hyperlipidemia (18), male sex and smoking (19), have also been reported, which is in agreement with our results. Association between arterial healing after dissections and FMD or other atherogenic risk factors suggest that NO-dependent endothelial function might have an effect on healing from a macroscopic injury, as it does on healing from oxidative injury (20). In contrast to NO-mediated suppression of cellular processes, endothelial dysfunction results in reactive oxygen species-mediated cellular activation and dysfunctional arterial repair. Furthermore, mobilization of the circulating endothelial progenitor cells is also partly NO-dependent (20). Our study results may be impetus for targeted therapy to enhance endothelial function in order to promote arterial healing and reduce future complications and medical expenses.

While there was a tendency for higher PWV to be associated with older age or steno-occlusive patterns, it was associated with ischemic presentation even after controlling for such variables. This is in agreement with previous literature that has reported higher levels of PWV in ischemic stroke patients compared to age and sex matched patients admitted for medical diseases (21). There may be possible explanations. First, increases in central aortic pulse pressure have been shown to be inter-related with thrombogenicty, as measured by thrombin-induced platelet-fibrin clot length (22). Thus, even with similar dissecting arterial segments, risk for stroke in individual patients may differ according to their thrombogenicity. This might have been represented by higher PWV tertiles in the current study. Second, there is a chance that central arterial stiffness will cause increased transmission of damaging pulsatile forces to the peripheral vasculature, resulting in propagation of the dissecting segment (23). Propagation of the dissection segment will in turn result in increased chance of occlusion of perforators, branching arteries, or thromboembolism (24). The exact underlying pathomechanism, and whether PWV is associated with ischemic stroke recurrence, needs to be confirmed in future studies.

There are some issues regarding the method of measurement of endothelial function and central arterial stiffness in this study that deserves attention. In this study, endothelial function was measured by brachial FMD, and considered a surrogate of cerebral endothelial function. Brachial FMD is known to be associated with coronary endothelial function (25), and is likely to represent arterial healing capacity of global arterial bed (20). However, there is controversy as to whether brachial FMD may adequately reflect endothelial function of cranial vessels, as literature comparing L-arginine induced cerebrovascular reactivity and brachial FMD showed low correlation (26). Currently, there are various methods of assessment of cerebral endothelial function (27), and there is no clear consensus how best to evaluate cerebral endothelial function, and future study is needed in this regard. It should be also noted that in this study, brachial-ankle PWV was interpreted as an index of central arterial stiffness. The carotid-femoral PWV is considered the gold standard of noninvasive measurement of arterial stiffness (7). Brachial-ankle PWV is known to be well-correlated with carotid-femoral PWV, but still some portions of brachial-ankle PWV may be determined by peripheral arterial stiffness, and may have affected the results of the current study (28).

This study has some limitations. First, due to the ethnic characteristics of the location where the study was performed, there was a high rate of intracranial dissections as compared to extracranial dissections. Apart from anatomical differences of arterial wall involvement (29), reports have shown that risk factors such as hypertension or minor trauma differ according to extracranial versus intracranial involvement (30). Accordingly, there is a chance that the main findings of the current study may be more significant in intracranial dissections. It should be also taken into consideration that extracranial to intracranial extended dissections were considered to be extracranial dissections in the current study. Second, although the institutional CCAD registry included all consecutive patients irrespective of subspecialty mainly involved in the patient's management, there may have been selection bias, for patients that underwent FMD and PWV were selected for analysis. Indeed, there was a tendency for ischemic presentations in patients that was admitted to neurology, while patients with concerns for aneurysmal rupture were admitted to neurosurgery. In patients admitted to the neurology department, FMD & PWV was omitted in patients with severe stroke, or patients that presented without definite neurologic deficits. However, a substantial number of dilatation morphologies were included in the analysis, which strengthens the generalizability of association between FMD and arterial healing. The predictive ability of FMD as a biomarker of spontaneous arterial healing will need to be confirmed in future studies. The finding of an association between PWV and ischemic presentations might need further validation in a cohort with a more even distribution of hemorrhagic and ischemic presentations. Third, the treatment methods were heterogeneous, with some patients undergoing early interventional or surgical repair, while medical regimens also differed. The results of the CADISS trial reported no differences in stroke recurrence or arterial healing following antiplatelet or anticoagulant medication in cases with extracranial dissections (31): however, this has not been validated for intracranial dissections, which constituted a large number of our patients. Further studies are necessary to address this issue. Fourth, use of FMD as a single-measurement screening test with predefined cut-off points to define endothelial dysfunction may be limited by its inter-individual variability of measurements (32). Future studies are warranted using more sensitive and non-operator dependent indexes such as reactive hyperemia index (33–35) or shear wave elastography (36). In the meanwhile, the role of brachial FMD and brachial-arterial PWV as a practical bedside tool for management of CCAD patients also need to the further addressed.

In conclusion, mechanical and physiological properties of the arterial wall significantly affect the clinical behavior of spontaneous CCAD. A preserved endothelial function may promote arterial healing, while increased arterial stiffness may be associated with increased ischemic presentation.

## Data availability statement

The raw data supporting the conclusions of this article will be made available by the authors, without undue reservation.

## Ethics statement

The studies involving human participants were reviewed and approved by Ajou University Hospital IRB. Written informed consent for participation was not required for this study in accordance with the national legislation and the institutional requirements.

## Author contributions

S-JL: data interpretation, drafted the work, revised the draft critically for important intellectual content, conceptualization and supervision of the study, and approved the final version of the paper. JL, MK, SP, WJ, JC, and JH: data acquisition, data interpretation, revised the draft critically for important intellectual content, and approved the final version of the paper. JP and BP: data interpretation, revised the draft critically for important intellectual content. All authors contributed to the article and approved the submitted version.

## Funding

This work was supported by the new faculty research fund of the Ajou University School of Medicine and by the Basic Science Research Program through the National Research Foundation of Korea (NRF) funded by the Ministry of Education (NRF-2021R1I1A1A01048331; S-JL).

## Conflict of interest

The authors declare that the research was conducted in the absence of any commercial or financial relationships that could be construed as a potential conflict of interest.

## Publisher's note

All claims expressed in this article are solely those of the authors and do not necessarily represent those of their affiliated organizations, or those of the publisher, the editors and the reviewers. Any product that may be evaluated in this article, or claim that may be made by its manufacturer, is not guaranteed or endorsed by the publisher.

## Abbreviations

CCAD: cervicocephalic arterial dissection
FMD: flow-mediated dilation
PWV: pulse wave velocity
NIHSS: National Institute of Health Stroke Scale
mRS: modified Rankin Scale.

## References

[1] Fusco MR Harrigan MR. Cerebrovascular dissections–a review part i: spontaneous dissections. Neurosurgery. (2011) 68:242–57; discussion 57. 10.1227/NEU.0b013e3182012323 21150764

[2] Ahn SS Kim BM Suh SH Kim DJ Kim DI Shin YS. Spontaneous symptomatic intracranial vertebrobasilar dissection: initial and follow-up imaging findings. Radiology. (2012) 264:196–202. 10.1148/radiol.12112331 22550310

[3] Horio Y Ogata T Abe H Fukuda K Morishita T Higashi T. Factors predictive of enlargement of dissecting aneurysms in the vertebral artery. World Neurosurg. (2021) 151:e935–e42. 10.1016/j.wneu.2021.05.024 34020061

[4] Inagami T Naruse M Hoover R. Endothelium as an endocrine organ. Annu Rev Physiol. (1995) 57:171–89. 10.1146/annurev.ph.57.030195.001131 7778863

[5] Van Belle E Bauters C Asahara T Isner JM. Endothelial regrowth after arterial injury: from vascular repair to therapeutics. Cardiovasc Res. (1998) 38:54–68. 10.1016/S0008-6363(97)00326-X 9683907

[6] Celermajer DS Sorensen KE Gooch VM Spiegelhalter DJ Miller OI Sullivan ID. Non-invasive detection of endothelial dysfunction in children and adults at risk of atherosclerosis. Lancet. (1992) 340:1111–5. 10.1016/0140-6736(92)93147-F 1359209

[7] Della Corte V Tuttolomondo A Pecoraro R Di Raimondo D Vassallo V Pinto A. Inflammation, endothelial dysfunction and arterial stiffness as therapeutic targets in cardiovascular medicine. Curr Pharm Des. (2016) 22:4658–68. 10.2174/1381612822666160510124801 27160758

[8] Maida CD Vasto S Di Raimondo D Casuccio A Vassallo V Daidone M. Inflammatory activation and endothelial dysfunction markers in patients with permanent atrial fibrillation: a cross-sectional study. Aging. (2020) 12:8423–33. 10.18632/aging.103149 32364529 PMC7244079

[9] Ross R. The pathogenesis of atherosclerosis: a perspective for the 1990s. Nature. (1993) 362:801–9. 10.1038/362801a0 8479518

[10] Kim HL Kim SH. Pulse wave velocity in atherosclerosis. Front Cardiovasc Med. (2019) 6:41. 10.3389/fcvm.2019.00041 31024934 PMC6465321

[11] Bruno RM Bianchini E Faita F Taddei S Ghiadoni L. Intima media thickness, pulse wave velocity, and flow mediated dilation. Cardiovasc Ultrasound. (2014) 12:34. 10.1186/1476-7120-12-34 25148901 PMC4154051

[12] Debette S Compter A Labeyrie MA Uyttenboogaart M Metso TM Majersik JJ. Epidemiology, pathophysiology, diagnosis, and management of intracranial artery dissection. Lancet Neurol. (2015) 14:640–54. 10.1016/S1474-4422(15)00009-5 25987283

[13] Lee SJ Hong JM Lee SE Kang DR Ovbiagele B Demchuk AM. Association of fibrinogen level with early neurological deterioration among acute ischemic stroke patients with diabetes. BMC Neurol. (2017) 17:101. 10.1186/s12883-017-0865-7 28525972 PMC5438529

[14] Kleindorfer DO Towfighi A Chaturvedi S Cockroft KM Gutierrez J Lombardi-Hill D. 2021 guideline for the prevention of stroke in patients with stroke and transient ischemic attack: a guideline from the American Heart Association/American Stroke Association. Stroke. (2021) 52:e364–467. 10.1161/STR.0000000000000375 34024117

[15] Shin DH Lee JS Hong JM Kim SY. Cross-section imaging with m-Mode as an alternative method for the measurement of brachial artery flow-mediated vasodilation. J Clin Ultrasound. (2013) 41:158–63. 10.1002/jcu.21972 22811368

[16] Maruhashi T Kajikawa M Kishimoto S Hashimoto H Takaeko Y Yamaji T. Diagnostic criteria of flow-Mediated vasodilation for normal endothelial function and nitroglycerin-induced vasodilation for normal vascular smooth muscle function of the brachial artery. J Am Heart Assoc. (2020) 9:e013915. 10.1161/JAHA.119.013915 31910779 PMC7033833

[17] Kim J Song TJ Kim EH Lee KJ Lee HS Nam CM. Brachial-Ankle pulse wave velocity for predicting functional outcome in acute stroke. Stroke. (2014) 45:2305–10. 10.1161/STROKEAHA.114.005576 24968933

[18] Daou B Hammer C Chalouhi N Starke RM Jabbour P Rosenwasser RH. Dissecting pseudoaneurysms: predictors of symptom occurrence, enlargement, clinical outcome, and treatment. J Neurosurg. (2016) 125:936–42. 10.3171/2015.10.JNS151846 26824374

[19] Kim MK Lim YC. Conservative management of unruptured spontaneous intracranial vertebral artery dissection. World Neurosurg. (2019) 126:e402–9. 10.1016/j.wneu.2019.02.063 30822585

[20] Deanfield JE Halcox JP Rabelink TJ. Endothelial function and dysfunction: testing and clinical relevance. Circulation. (2007) 115:1285–95. 10.1161/CIRCULATIONAHA.106.652859 17353456

[21] Tuttolomondo A Casuccio A Della Corte V Maida C Pecoraro R Di Raimondo D. Endothelial function and arterial stiffness indexes in subjects with acute ischemic stroke: relationship with toast subtype. Atherosclerosis. (2017) 256:94–9. 10.1016/j.atherosclerosis.2016.10.044 27817840

[22] Chen G Bliden KP Chaudhary R Liu F Kaza H Navarese EP. Central aortic pulse pressure, thrombogenicity and cardiovascular risk. J Thromb Thrombolysis. (2017) 44:223–33. 10.1007/s11239-017-1524-y 28695310

[23] Yu S McEniery CM. Central versus peripheral artery stiffening and cardiovascular risk. Arterioscler Thromb Vasc Biol. (2020) 40:1028–33. 10.1161/ATVBAHA.120.313128 32188277

[24] Matsukawa H Shinoda M Fujii M Takahashi O Uemura A Niimi Y. Basilar extension and posterior inferior cerebellar artery involvement as risk factors for progression of the unruptured spontaneous intradural vertebral artery dissection. J Neurol Neurosurg Psychiatry. (2014) 85:1049–54. 10.1136/jnnp-2013-306931 24463481

[25] Anderson TJ Uehata A Gerhard MD Meredith IT Knab S Delagrange D. Close relation of endothelial function in the human coronary and peripheral circulations. J Am Coll Cardiol. (1995) 26:1235–41. 10.1016/0735-1097(95)00327-4 7594037

[26] Pretnar-Oblak J Sabovic M Zaletel M. Associations between systemic and cerebral endothelial impairment determined by cerebrovascular reactivity to l-Arginine. Endothelium. (2007) 14:73–80. 10.1080/10623320701346692 17497363

[27] Stevenson SF Doubal FN Shuler K Wardlaw JM. A systematic review of dynamic cerebral and peripheral endothelial function in lacunar stroke versus controls. Stroke. (2010) 41:e434–42. 10.1161/STROKEAHA.109.569855 20395619

[28] Sugawara J Hayashi K Yokoi T Cortez-Cooper MY DeVan AE Anton MA. Brachial-Ankle pulse wave velocity: an index of central arterial stiffness? J Hum Hypertens. (2005) 19:401–6. 10.1038/sj.jhh.1001838 15729378

[29] de Bray JM Penisson-Besnier I Dubas F Emile J. Extracranial and intracranial vertebrobasilar dissections: diagnosis and prognosis. J Neurol Neurosurg Psychiatry. (1997) 63:46–51. 10.1136/jnnp.63.1.46 9221967 PMC2169649

[30] Shin DH Hong JM Lee JS Nasim R Sohn SI Kim SJ. Comparison of potential risks between intracranial and extracranial vertebral artery dissections. Eur Neurol. (2014) 71:305–12. 10.1159/000357867 24662973

[31] Markus HS Levi C King A Madigan J Norris J Cervical Artery Dissection in Stroke Study (CADISS) Investigators. Antiplatelet therapy vs anticoagulation therapy in cervical artery dissection: the cervical artery dissection in stroke study (Cadiss) randomized clinical trial final results. JAMA Neurol. (2019) 76:657–64. 10.1001/jamaneurol.2019.0072 30801621 PMC6563567

[32] Sejda T Pit'ha J Svandova E Poledne R. Limitations of non-Invasive endothelial function assessment by brachial artery flow-mediated dilatation. Clin Physiol Funct Imaging. (2005) 25:58–61. 10.1111/j.1475-097X.2004.00590.x 15659082

[33] Tuttolomondo A Cirrincione A Casuccio A Del Cuore A Daidone M Di Chiara T. Efficacy of dulaglutide on vascular health indexes in subjects with type 2 diabetes: a randomized trial. Cardiovasc Diabetol. (2021) 20:1. 10.1186/s12933-020-01183-5 33397395 PMC7784355

[34] Tuttolomondo A Di Raimondo D Casuccio A Guercio G Del Cuore A Puleo MG. Endothelial function, adipokine serum levels and white matter hyperintesities in subjects with diabetic foot syndrome. J Clin Endocrinol Metab. (2019) 104:3920–30. 10.1210/jc.2018-02507 30977833

[35] Tuttolomondo A Petta S Casuccio A Maida C Corte VD Daidone M. Reactive Hyperemia Index (RHI) and cognitive performance indexes are associated with histologic markers of liver disease in subjects with Non-Alcoholic Fatty Liver Disease (NAFLD): a Case control study. Cardiovasc Diabetol. (2018) 17:28. 10.1186/s12933-018-0670-7 29452601 PMC5815178

[36] Gulsen F Samanci C Memis Durmaz ES Durmaz E Tel C Gencturk M. Brachial artery wall stiffness assessment by shear wave elastography: a Promising new diagnostic tool for endothelial dysfunction detection. J Ultrasound Med. (2018) 37:1977–83. 10.1002/jum.14548 29363817

