# Supplementary material for: Influence of endothelial function and arterial stiffness on the behavior of cervicocephalic arterial dissections: An observational study
Source: Front Neurol. 2022 Aug 29;13:968488. doi: 10.3389/fneur.2022.968488 (PMC9464973; doi:10.3389/fneur.2022.968488)
Supplement: Supplementary file 1 [file Data_Sheet_1.docx]

**Influence of endothelial function and arterial stiffness on the behavior of cervicocephalic arterial dissections: an observational study**

**Supplementary Table 1.** Comparison of baseline profiles, treatment, clinical and imaging characteristics of patients that were treated for the acute management of cervicocephalic arterial dissections according to disposition and availability of FMD & PWV data.

| **Disposition** | **Neurology** | | **Neurosurgery** |  |  |
| --- | --- | --- | --- | --- | --- |
|  | **FMD&PWV (+)**  **(N=146)** | **FMD&PWV (-)**  **(N=120)** | **(N=51)** | **P-value*** | **P-value†** |
| Age | 47 ± 11 | 45 ± 12 | 47 ± 10 | 0.396 | 0.607 |
| Sex, male | 113 (77.4%) | 72 (60.0%) | 33 (64.7%) | 0.008 | 0.002 |
| Presentation pattern |  |  |  | <0.001 | 0.002 |
| *Ischemic* | 112 (76.7%) | 71 (59.2%) | 8 (15.7%) |  |  |
| *Headache, others* | 34 (23.3%) | 49 (40.8%) | 43 (84.3%) |  |  |
| Morphology |  |  |  | <0.001 | 0.035 |
| *Stenosis* | 37 (25.3%) | 40 (33.3%) | 6 (11.8%) |  |  |
| *Dilatation* | 48 (32.9%) | 48 (40.0%) | 43 (84.3%) |  |  |
| *Occlusion* | 61 (41.8%) | 32 (26.7%) | 2 (3.9%) |  |  |
| Circulation |  |  |  | 0.123 | 0.352 |
| *Carotid* | 44 (30.1%) | 30 (25.0%) | 8 (15.7%) |  |  |
| *Vertebro-basilar* | 102 (69.9%) | 90 (75.0%) | 43 (84.3%) |  |  |
| Intra vs. extracranial |  |  |  | <0.001 | 0.251 |
| *Intracranial* | 95 (65.1%) | 86 (71.7%) | 48 (94.1%) |  |  |
| *Extracranial* | 51 (34.9%) | 34 (28.3%) | 3 (5.9%) |  |  |
| HTN | 58 (39.7%) | 38 (31.7%) | 9 (17.6%) | 0.014 | 0.173 |
| DM | 12 (8.2%) | 11 (9.2%) | 3 (5.9%) | 0.774 | 0.784 |
| Smoking | 60 (41.1%) | 39 (32.5%) | 15 (29.4%) | 0.197 | 0.149 |
| Dyslipidemia | 33 (22.6%) | 25 (20.8%) | 2 (3.9%) | 0.011 | 0.728 |
| Treatments |  |  |  | 0.011 | 0.833 |
| *Medical* | 128 (87.7%) | 106 (88.3%) | 34 (66.7%) |  |  |
| *Acute intervention* | 15 (10.3%) | 12 (10.0%) | 15 (29.4%) |  |  |
| *Delayed intervention* | 1 (0.7%) | 0 (0.0%) | 1 (2.0%) |  |  |
| *Bypass operation* | 2 (1.4%) | 2 (1.7%) | 1 (2.0%) |  |  |
| Medications |  |  |  | <0.001 | 0.018 |
| *None* | 8 (5.5%) | 17 (14.2%) | 14 (27.5%) |  |  |
| *Antiplatelet* | 51 (34.9%) | 48 (40.0%) | 36 (70.6%) |  |  |
| *Anticoagulation* | 87 (59.6%) | 55 (45.8%) | 1 (2.0%) |  |  |
| **Clinical follow-up** | (N=143) | (N=113) | (N=47) |  |  |
| 3-month mRS | 1..0 [0.0 – 2.0] | 1..0 [0.0 – 2.0] | 1.0 [0.0 – 1.0] | 0.164 | 0.607 |
| New ischemic stroke | 8 (5.6%) | 2 (1.8%) | 0 (0.0%) | 0.091 | 0.117 |
| New SAH | 0 (0.0%) | 1 (0.9%) | 0 (0.0%) | 0.430 | 0.260 |
| **Image follow-up** | (N=129) | (N=93) | (N=30) |  |  |
| Spont. arterial healing | 72 (55.8%) | 42 (45.2%) | 17 (56.7%) | 0.209 | 0.117 |
| Aneurysm enlargement | 4 (3.1%) | 4 (4.3%) | 3 (10.0%) | 0.249 | 0.636 |
| **Ischemic course** | (N=112) | (N=71) | (N=8) |  |  |
| NIHSS | 2.0 [1.0 – 5.0] | 4.0 [1.0 – 9.0] | 2.5 [1.25 – 10.5] | 0.079 | 0.094 |
| END | 13 (11.6%) | 10 (14.1%) | 0 (0.0%) | 0.498 | 0.589 |
| 3-month mRS | 1.0 [0.0 – 2.0] | 1.0 [0.0 – 3.0] | 1.0 [1.0 – 3.5] | 0.073 | 0.096 |

* FMD&PWV (+) vs. FMD&PWV (-) vs. Neurosurgery.

†FMD&PWV (+) vs. FMD&PWV (-).

FMD, flow mediated dilatation; PWV, pulse wave velocity; HTN, hypertension; DM, diabetes mellitus; mRS, modified Rankin Scale; SAH, subarachnoid hemorrhage; NIHSS, National Institute of Health Stroke Scale; END, early neurological deterioration.

**Supplementary Table 2.** Comparison of baseline profiles, treatment, clinical and imaging characteristics of included patients according to status of arterial healing.

|  | **Arterial**  **healing (-)**  **(N=57)** | **Arterial**  **healing (+)**  **(N=72)** | **P-value** |
| --- | --- | --- | --- |
| Age | 50 ± 12 | 45 ± 10 | 0.010 |
| Sex, male | 51 (70.8%) | 49 (86.0%) | 0.041 |
| Presentation pattern |  |  | 0.954 |
| *Ischemic* | 43 (75.4%) | 54 (75.0%) |  |
| *Headache, others* | 18 (25.0%) | 14 (24.6%) |  |
| Morphology |  |  | 0.224 |
| *Stenosis* | 17 (29.8%) | 13 (18.1%) |  |
| *Dilatation* | 16 (28.1%) | 28 (38.9%) |  |
| *Occlusion* | 24 (42.1%) | 31 (43.1%) |  |
| Circulation |  |  | 0.611 |
| *Carotid* | 19 (33.3%) | 21 (29.2%) |  |
| *Vertebro-basilar* | 38 (66.7%) | 51 (70.8%) |  |
| Intra vs. extracranial |  |  | 0.218 |
| *Intracranial* | 40 (70.2%) | 43 (59.7%) |  |
| *Extracranial* | 17 (29.8%) | 29 (40.3%) |  |
| HTN | 25 (43.9%) | 27 (37.5%) | 0.465 |
| DM | 8 (14.0%) | 1 (1.4%) | 0.005 |
| Smoking | 24 (42.1%) | 27 (37.5%) | 0.595 |
| Dyslipidemia | 12 (21.1%) | 17 (23.6%) | 0.730 |
| Treatments |  |  | 0.101 |
| *Medical* | 53 (93.0%) | 59 (81.9%) |  |
| *Acute intervention* | 3 (5.3%) | 11 (15.3%) |  |
| *Delayed intervention* | 1 (1.8%) | 0 (0.0%) |  |
| *Bypass operation* | 0 (0.0%) | 2 (2.8%) |  |
| Medications |  |  | 0.478 |
| *None* | 2 (3.5%) | 4 (5.6%) |  |
| *Antiplatelet* | 23 (40.4%) | 22 (30.6%) |  |
| *Anticoagulation* | 32 (56.1%) | 46 (63.6%) |  |
| FMD, mean | 6.1 ± 1.7% | 7.1 ± 1.8% | <0.001 |
| FMD, dichotomized |  |  | <0.001 |
| *Normal* | 24 (42.1%) | 57 (79.2%) |  |
| *Decreased* | 33 (57.9%) | 15 (20.8%) |  |
| PWV, mean | 1537 ± 337 | 1457 ± 279 | 0.316 |
| PWV, trichotomized |  |  | 0.272 |
| *1st tertile* | 19 (33.33) | 25 (34.72) |  |
| *2nd tertile* | 16 (28.07) | 28 (38.89) |  |
| *3rd tertile* | 22 (38.6) | 19 (26.39) |  |
| **Clinical follow-up** |  |  |  |
| 3-month mRS | 2.0 [1.0 – 3.75] | 1.0 [0.0 – 1.75] | 0.822 |
| New ischemic stroke | 4 (7.0%) | 4 (5.6%) | 0.732 |
| New SAH | 0 (0.0%) | 0 (0.0%) |  |
| **Image follow-up** |  |  |  |
| Aneurysm enlargement | 3 (5.3%) | 1 (1.4%) | 0.207 |
| **Ischemic course** | (N=43) | (N=54) |  |
| NIHSS | 2.0 [1.0 – 4.0] | 3.0 [0.0 – 5.5] | 0.708 |
| END | 4 (9.3%) | 9 (16.7%) | 0.290 |
| 3-month mRS | 1.0 [0.0 – 2.0] | 1.0 [0.75 – 2.0] | 0.209 |

HTN, hypertension; DM, diabetes mellitus; FMD, flow mediated dilatation; PWV, pulse wave velocity; mRS, modified Rankin Scale; SAH, subarachnoid hemorrhage; NIHSS, National Institute of Health Stroke Scale; END, early neurological deterioration.

**Supplementary Table 3.** Comparison of baseline profiles, treatment, clinical and imaging characteristics of included patients according to clinical presentation.

|  | **Headache & others**  **(N=34)** | **Ischemic stroke**  **(N=112)** | **P-value** |
| --- | --- | --- | --- |
| Age | 47 ± 10 | 47 ± 11 | 0.860 |
| Sex, male | 21 (61.8%) | 92 (82.1%) | 0.013 |
| Morphology |  |  | <0.001 |
| *Stenosis* | 8 (23.5%) | 29 (25.9%) |  |
| *Dilatation* | 20 (58.8%) | 28 (25.0%) |  |
| *Occlusion* | 6 (17.7%) | 55 (49.1%) |  |
| Circulation |  |  | 0.043 |
| *Carotid* | 5 (14.7%) | 39 (34.8%) |  |
| *Vertebro-basilar* | 29 (85.3%) | 73 (65.2%) |  |
| Intra vs. extracranial |  |  | 0.002 |
| *Intracranial* | 30 (88.2%) | 65 (58.0%) |  |
| *Extracranial* | 4 (11.8%) | 47 (42.0%) |  |
| HTN | 9 (26.5%) | 49 (43.8%) | 0.109 |
| DM | 2 (5.9%) | 10 (8.9%) | 0.733 |
| Smoking | 10 (29.4%) | 50 (44.6%) | 0.167 |
| Dyslipidemia | 3 (8.8%) | 30 (26.8%) | 0.05 |
| Treatments |  |  | 0.015 |
| *Medical* | 33 (97.1%) | 95 (84.8%) |  |
| *Acute intervention* | 0 (0.0%) | 15 (13.4%) |  |
| *Delayed intervention* | 1 (2.9%) | 0 (0.0%) |  |
| *Bypass operation* | 0 (0.0%) | 2 (1.8%) |  |
| Medications |  |  | 0.670 |
| *None* | 1 (2.9%) | 7 (6.3%) |  |
| *Antiplatelet* | 11 (32.4%) | 40 (35.7%) |  |
| *Anticoagulation* | 22 (64.7%) | 65 (58.0%) |  |
| FMD, mean | 6.9 ± 2.1% | 6.7 ± 1.9% | 0.662 |
| FMD, dichotomized |  |  | 0.340 |
| *Decreased* | 10 (29.4%) | 43 (38.4%) |  |
| *Normal* | 24 (70.6%) | 69 (61.6%) |  |
| PWV, mean | 1349 ± 180 | 1567 ± 411 | <0.001 |
| PWV, trichotomized |  |  | 0.001 |
| *1st tertile* | 17 (50.0%) | 32 (28.6%) |  |
| *2nd tertile* | 15 (44.1%) | 35 (31.3%) |  |
| *3rd tertile* | 2 (5.9%) | 45 (40.2%) |  |
| **Clinical follow-up** | (N=33) | (N=110) |  |
| 3-month mRS | 0.0 [0.0- 1.0] | 1.0 [0.0 – 2.0] | <0.001 |
| New ischemic stroke | 0 (0.0%) | 8 (7.3%) | 0.111 |
| New SAH | 0 (0.0%) | 0 (0.0%) |  |
| **Image follow-up** | (N=32) | (N=97) |  |
| Spont. Arterial healing | 18 (56.3%) | 54 (55.7%) | 0.954 |
| Aneurysm enlargement | 2 (6.3%) | 2 (2.1%) | 0.236 |

HTN, hypertension; DM, diabetes mellitus; FMD, flow mediated dilatation; PWV, pulse wave velocity; mRS, modified Rankin Scale; SAH, subarachnoid hemorrhage; NIHSS, National Institute of Health Stroke Scale; END, early neurological deterioration.
